# Editorial: Cancer Metabolism: Current Knowledge and Perspectives

**DOI:** 10.3389/fonc.2019.00287

**Published:** 2019-04-16

**Authors:** Leonardo Freire-de-Lima, Lucia Mendonça-Previato, Luciana Boffoni Gentile

**Affiliations:** ^1^Laboratorio de Glicobiologia, Instituto de Biofisica Carlos Chagas Filho, Universidade Federal do Rio de Janeiro, Rio de Janeiro, Brazil; ^2^Laboratório de Patologia Clínica Veterinária, Hospital Universitário de Medicina Veterinária Professor Firmino Mársico Filho (Huvet), Universidade Federal Fluminense, Rio de Janeiro, Brazil

**Keywords:** cancer, metabolism, glycolysis (glycolytic pathway), multidrug resistance phenotype, invasiviness

Over the first half of the twentieth century, it has been demonstrated that an important hallmark of cancer cells is the reprogramming of cellular metabolism. The capacity to obtain nutrients from an extremely poor microenvironment and utilize it to both generate biomass and maintain the cellular viability is a major feature of transformed cells. Several papers have proposed new mechanisms by which cancer cells undergo substantial metabolic reprogramming to endure within the hostile microenvironment. However, the metabolic profile adopted by cancer cells with invasive and multidrug-resistance phenotypes is still poorly understood. The research topic “Cancer Metabolism: Current Knowledge and Perspectives” provides exciting articles on the valuable field of cancer metabolism, where one hypothesis and theory, two original researches and five outstanding reviews are presented.

In their original research, Herrero and Gutierrez demonstrated that multiple myeloma cells with ongoing endogenous DNA damage depend on a homologous recombination (HR) pathway, which may be used as therapeutic proposals. They suggested that obstructing HR after the initial step of end resection might be more suitable to promote cell death, since it avoids a compensatory non-homologous end joining repair mechanism. The authors claim that these preclinical observations afford the basis for its clinical assessment.

Sant'Anna-Silva et al. analyzed the metabolic changes to the metastatic phenotype of human tongue squamous cell carcinoma lineages. By using both metabolomic and fluorescence lifetime imaging microscopy analysis, the authors demonstrated that many pathways linked to lipid metabolism seem to be associated to metastatic phenotype. On the other hand, amino acid metabolism and cell cycle regulation are most correlated to cells with low invasive phenotype.

In their review article, Turgeon et al. addressed evidence that link DNA damage/repair mechanisms with cancer cell metabolism. The authors claim that such connection is progressively apparent, granting opportunities to better understand the metabolic susceptibilities of a considerable fraction of tumors.

Prakasam et al., summarized recent progress in the understanding of many posttranslation modifications (PTMs) in cancer, in particular the PTMs in the M2 isoform of pyruvate kinase (PKM2). The authors believe that such knowledge will be crucial to evaluate their therapeutic potential for the treatment of different types of cancer.

Morrot et al. provided a snapshot of metabolic reprogramming in cancer cells, describing how, even in aerobic conditions, transformed cells opt for glycolysis instead of oxidative phosphorylation (OXPHOS). They discussed how this metabolic reprogramming is able to induce a high-lactate output, then promoting mmunosuppressive events. The authors believe that further studies are needed to better understand the effect of lactate and other “waste” metabolites on cancer progression.

Coelho et al. presented an overview of the metabolic reprogramming in thyroid cancer, emphasizing factors that promote enhanced glycolysis in transformed cells. The authors also discussed about promising metabolic targets that might be useful to treat patients with thyroid cancer.

Snyder et al. discussed about the metabolic phenotype of cancer stem cells (CSCs), especially taking into account both glycolytic and OXPHOS pathways. Since CSCs present metabolic peculiarities when compared to most of cancer cells in a tumor, the authors believe that such singularities might offer a great potential for developing improved treatments for cancer patients.

Finally, in their hypothesis and theory article, Vidal et al. addressed the connection between multidrug-resistance phenotype and metabolic reprogramming in cancer cells, taking into consideration the functions mediated by ATP-binding cassette transporters, as well as the numerous non-metabolic roles mediated by enzymes that are part of the glycolytic pathway, with special attention to glyceraldehyde-3-phosphate dehydrogenase.

Taken together, the published papers in this research topic strengthen the concept that cancer cell metabolism is an important field in cancer biology, and further studies in this lively research area might provide important information, which may be useful for treatment, diagnostic and therapeutic purposes.

## Author Contributions

LF, LM-P and LG wrote the paper. All the authors read and approved the final version of the manuscript.

### Conflict of Interest Statement

The authors declare that the research was conducted in the absence of any commercial or financial relationships that could be construed as a potential conflict of interest.

